# Interfacing non-enzymatic catalysis with living microorganisms

**DOI:** 10.1039/d1cb00072a

**Published:** 2021-06-04

**Authors:** Joanna C. Sadler, Jonathan A. Dennis, Nick W. Johnson, Stephen Wallace

**Affiliations:** Institute of Quantitative Biology, Biochemistry and Biotechnology, School of Biological Sciences, University of Edinburgh Roger Land Building, Alexander Crum Brown Road, King's Buildings Edinburgh, EH9 3FF UK stephen.wallace@ed.ac.uk; School of Chemistry, University of Edinburgh Joseph Black Building, David Brewster Road, King's Buildings Edinburgh, EH9 3F UK

## Abstract

Interfacing non-enzymatic catalysis with cellular metabolism is emerging as a powerful approach to produce a range of high value small molecules and polymers. In this review, we highlight recent examples from this promising young field. Specifically, we discuss demonstrations of living cells mediating redox processes for biopolymer production, interfacing solar-light driven chemistry with microbial metabolism, and intra- and extracellular non-enzymatic catalysis to generate high value molecules. This review highlights the vast potential of this nascent field to bridge the two disciplines of synthetic chemistry and synthetic biology for a sustainable chemical industry.

## Abstract 2

This review highlights recent advances in the field of biocompatible chemistry. It focusses on the combined use of non-enzymatic catalysis and microbial metabolism to support cellular function and to synthesise high value chemicals.

## Graphical abstract

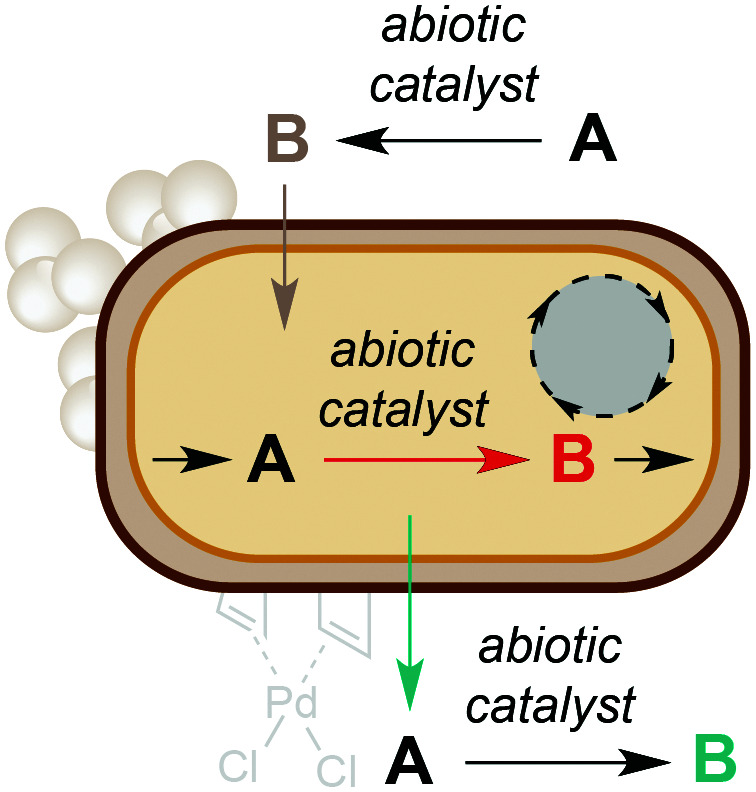

## 1. Introduction

Chemical catalysis is a powerful tool to efficiently access high value small molecules and materials. Compared to many biocatalysts, non-enzymatic catalysts offer wide substrate scope, high turnover numbers, and stability to a wide range of process conditions. However, chemical syntheses often rely on fossil fuel-derived starting materials which limits the long-term sustainability of this approach. Synthetic biology offers a promising alternative by accessing high value chemicals from low cost renewable or waste feedstocks under mild conditions.^1–3^ However, biological pathways are inherently limited by the reactions known to Nature, precluding access to large areas of chemical space. This has prompted researchers to explore opportunities in merging these two traditionally discrete disciplines to benefit from the best of both worlds.^4,5^

**Fig. 1:**
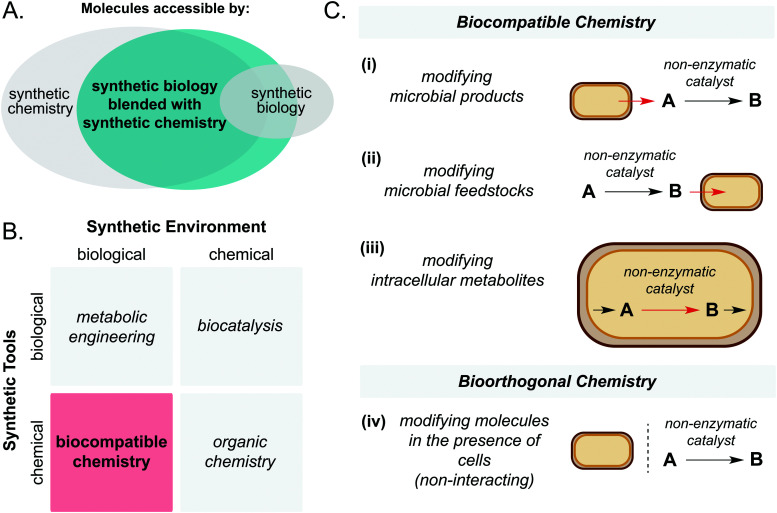
(A) Synthetic biology blended with synthetic chemistry enables access to larger areas of chemical space than synthetic biology alone. (B) Biocompatible chemistry involves the use of chemical synthetic tools in a biological synthetic environment. (C) Biocompatible chemistry refers to abiotic catalysis which is directly interfaced with cellular metabolism, whereas bioorthogonal chemistry does not interact directly with cellular metabolism.

Through the union of non-enzymatic catalysis and microbial metabolism, reaction sequences may be accessed that would not be possible using either technology in isolation (Fig. 1A and B). A key article by Wallace and Balskus in 2014 summarised early contributions and opportunities in this exciting field.^4^ Therefore, the purpose of the present article is to summarise progress in this area in the period 2014–2021. Specifically, the scope will include: (i) living cell mediated electron transfer processes, (ii) solar-light driven chemistry interfaced with microbial metabolism and (iii) intra- and extracellular non-enzymatic catalysis for the production of high value small molecules. Whilst the field of artificial metalloenzymes is acknowledged as a promising approach to widen the scope of synthetic biology, it is not considered to be ‘non-enzymatic’; therefore it falls outside the scope of this review and instead we direct the reader to recent review articles on the topic.^6–9^ Finally, we will outline possible future directions of this emerging field.

First, it is important to define the terms ‘biocompatible chemistry’ and ‘bioorthogonal chemistry’ in the context of this review. Biocompatible chemistry is defined as non-enzymatic catalysis interfaced with the metabolism of a living organism. This may operate in one or more of three ways: non-enzymatic modification of a microbial metabolite; non-enzymatic generation of a microbial substrate; or non-enzymatic modification of a metabolite within a metabolic pathway. In contrast, bioorthogonal chemistry is defined as non-enzymatic modification of a small molecule or biopolymer which occurs independently of cellular metabolism (Fig. 1C).

## 2. Living cell mediated redox processes

### 2.1. Microbe mediated polymerisation

**Fig. 2:**
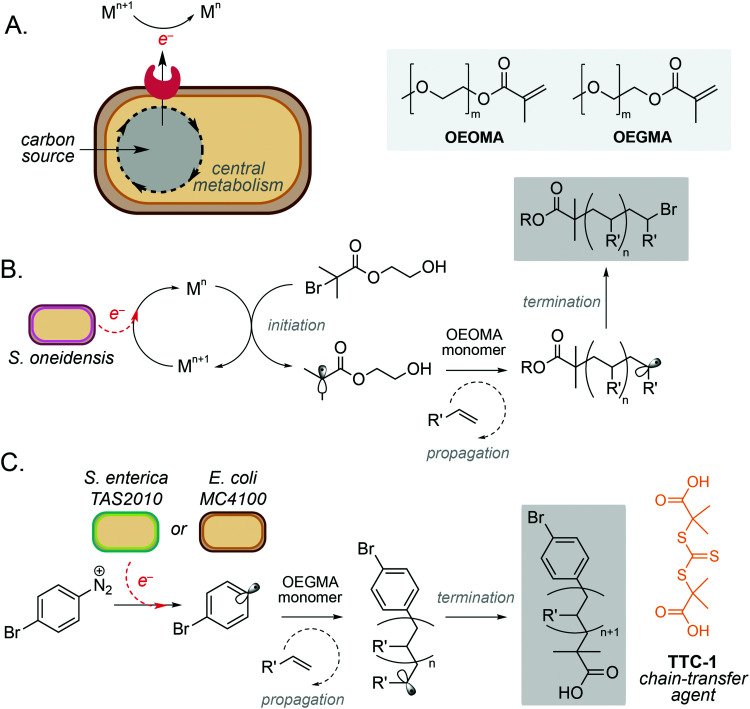
Microbe-mediated redox driven polymerisation reactions. (A) Extracellular electron transport proteins mediate redox processes using a wide range of metals and small molecules as electron acceptors. (B) Microbe mediated atom-transfer radical polymerisation for polymerisation of oligo(ethylene oxide) methyl ether methacrylate (OEOMA). (C) Controlled reversible addition–fragmentation chain transfer (RAFT) polymerisation of a oligo(ethylene glycol) methyl ether methacrylate (OEGMA) monomer using *E. coli* MC4100.

One of the simplest and most powerful applications of biocompatible chemistry is the use of metabolic electrons to catalyse abiotic reactions. This approach uses membrane-bound redox proteins found in obligate and facultative anaerobic microorganisms to drive non-enzymatic reactions at the cell surface (Fig. 2A). A key example is the use of microbe-initiated radical polymerisations to produce high value synthetic polymers. Such reactions avoid the use of stoichiometric reductants and harsh processing conditions typically required for this chemistry *in vitro*,^10,11^ and have the potential to create entirely new products through the controlled polymerisation of substrates within the architecture of the cell membrane.

Perhaps the most well studied of these is the Gram-negative anaerobic bacterium *Shewanella oneidensis*, which possesses extracellular electron transport (EET) machinery that can use a variety of molecules and metals as electron acceptors.^12–14^ This reactivity has recently been harnessed by Fan and co-workers, who demonstrated the use of *S. oneidensis* as ‘living electrodes’ in an atom transfer radical polymerisation (ATRP) of an oligo(ethylene oxide) methyl ether methacrylate (OEOMA) monomer under anaerobic conditions.^15^ Electrons from native respiratory pathways were able to reduce Cu(ii), Fe(ii) and Co(ii) catalysts to Cu(i), Fe(i) and Co(i), respectively, which could then react with a brominated initiator to produce a radical species and trigger olefin polymerisation (Fig. 2B).^15^ Further optimisation highlighted that a Cu(ii) tris(2-pyridylmethyl)amine (TPMA) complex was able to accelerate polymerisation and improve control of polymer chain length. The membrane-bound decaheme c-type cytochrome MtrC was found to be an essential electron donor to Cu(ii)-TPMA during the reaction, with a *S. oneidensis* Δ*mtrC* knockout strain demonstrating significantly decreased polymerisation rates. In a subsequent study, the authors demonstrated *S. oneidensis* mediated radical polymerisation under aerobic conditions.^16^ This is a significant achievement as radical polymerisation reactions are typically quenched by molecular oxygen. Interestingly, *S. oneidensis* was found to rapidly consume dissolved oxygen under polymerisation conditions *via* aerobic respiration, before directing extracellular electron flux to the metal catalyst at the membrane to initiate polymerisation. This natural ability to decrease oxygen levels enabled preparation of various synthetic polymers under aerobic conditions with comparable yields and chain lengths to those prepared under anaerobic conditions.^16^

Radical polymerisation has also been demonstrated using the metal-reducing Gram-negative bacterium *Cupriavidus metallidurans* and the model laboratory bacterium *Escherichia coli*.^17^ Native membrane reductases in both organisms were able to reduce Fe(iii) to Fe(ii) to activate a series of radical initiators for ATRP. Minimal polymerisation was observed in the absence of live cells, indicating the importance of cellular metabolism to the catalytic cycle to regenerate active Fe(ii).^17^ It is noteworthy that polymerisation was also observed with the Gram-positive anaerobe *Clostridium sporogenes*, however only 34% conversion was achieved in comparison to 61% and 78% for *C. metallidurans* and *E. coli*, respectively. This was hypothesised to be due to *C. sporogenes* being less efficient at reducing Fe(iii) than Gram-negative strains. The choice of ligand was also critical to the success of this reaction, with a balance between ligand dissociation, microbial toxicity and the overall rate of polymerisation being necessary to achieve optimum productivity and polydispersity.^15,17^ Finally, whilst this system focusses exclusively on the use and generation of water-soluble substrates and products, the integration of biphasic solvents systems or reaction compartmentalisation strategies could be used to broaden the scope of this approach, including the polymerisation of metabolic substrates generated from sustainable feedstocks.

More recently, microbe mediated polymerisation has also been demonstrated in the absence of a metal catalyst.^18^ In this seminal study, Nothling and co-workers demonstrated the reduction of an aryl diazonium initiator to generate a carbon-centred aryl radical species using the enteric Gram-negative pathogen *Salmonella enterica* serovar Typhimurium TAS2010 and the common laboratory bacterium *Escherichia coli* MC4100. This triggered controlled reversible addition–fragmentation chain transfer (RAFT) polymerisation of an oligo(ethylene glycol) methyl ether methacrylate (OEGMA) monomer using the trithiocarbonate chain-transfer agent, TTC-1 (Fig. 2C), with 100% monomer consumption in 24 hours. The reaction was shown to be initiated by redox-active shuttles in the cell membrane and facilitated by cellular metabolism. The initiator, 4-bromobenzenediazonium tetrafluoroborate (4-BT), was shown to be remarkably biocompatible at concentrations ≤800 μM, and both strains exhibited stationary phase redox potentials less than −200 mV (*vs.* Ag/AgCl) under aerobic and anaerobic conditions in a variety of growth media. Interestingly, both the cell membrane and intracellular reducing agents were required for maximum productivity, and the increased activity of *E. coli* MC4100 was attributed to favourable interactions between 4-BT and the cell membrane due to a decreased lipopolysaccharide (LPS) phenotype. Overall, this opens up exciting opportunities for laboratory strains of bacteria to participate in a wide range of metal-free abiotic polymerisation reactions to produce new sustainable materials and raises the intriguing possibility that diazo-containing natural products could initiate such reactivity in Nature.^18^

### 2.2. Harnessing microbial chemistry for solar light driven reactions

**Fig. 3:**
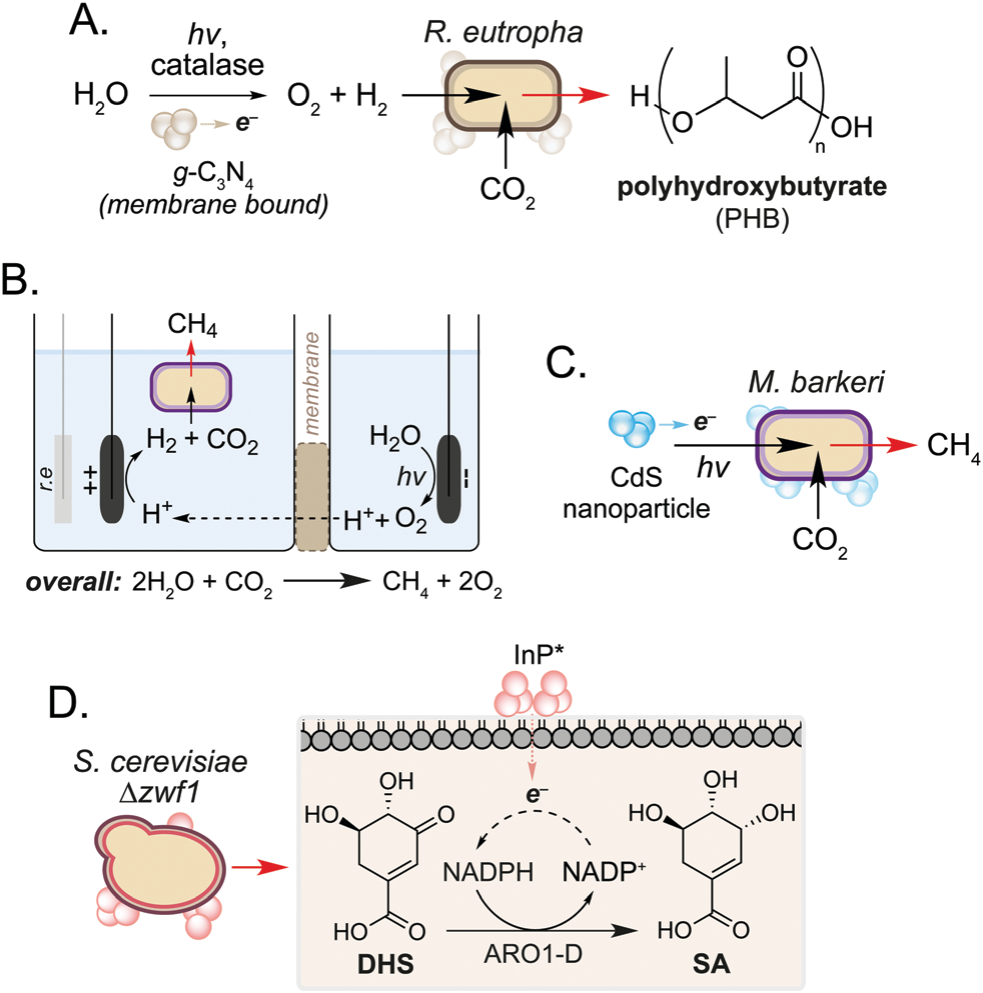
Biohybrid photosynthesis systems utilising solar-to-chemical energy conversion. (A) Use of a water-splitting photocatalyst for H_2_ production to increase PHB production in *R. eutropha*. (B) Two-compartment biocompatible electrochemical cell interfaced with a culture of living *M. barkeri* for biofuel production. r.e refers to the reference electrode. (C) CdS mediated photoexcited electron transfer for enhanced biofuel production in *M. barkeri*. (D) Biosynthesis of SA from DHS enabled by *in vivo* NADPH regeneration mediated by photogenerated electrons from InP nanoparticles.

In addition to use as a source of electrons, microorganisms can also accept electrons to drive cellular processes. Such systems are classical examples of microbial syntrophy,^19^ but more recently have also used biocompatible reactions to enable CO_2_ utilisation in an ongoing drive towards a sustainable, circular economy.^20–22^ Work in this field has focussed on hybrid photosynthesis systems that harness solar energy for H_2_ production from water.^23,24^ These use non-enzymatic photocatalysts which exhibit high solar-to-chemical energy conversion^24^ and have shown biocompatibility with various microorganisms.^25^ Seminal studies from Torella and co-workers and Liu and co-workers demonstrated the use of bioelectrochemical cells containing *R. eutropha* to produce PHB and fusal alcohols from CO_2_ and H_2_O.^26,27^ Using a reactive oxygen species (ROS)-resistant Co–P alloy cathode, the authors achieved CO_2_ reduction efficiencies exceeding that of natural photosynthesis.^26^ In a later study, Tremblay and co-workers interfaced a biocompatible graphitic carbon nitride (g-C_3_N_4_) photocatalyst and catalase-mediated H_2_O_2_ degradation with the facultative autotrophic Gram-negative bacterium *Ralstonia eutropha* to produce the biopolymer polyhydroxybutyrate (PHB) (Fig. 3A). When combined, the photocatalyst formed large cell membrane-associated aggregates which enabled charge transfer to intracellular hydrogenases in the presence of light, increasing H_2_ and O_2_ availability to the cell. This increased PHB titres from CO_2_ by 2.2-fold and 1.7-fold when g-C_3_N_4_/catalase and CdS nanorods were used, respectively.^28,29^

In a related study, the non-enzymatic production of H_2_ has been used to support methanogenesis in *Methanosarcina barkeri*, an autotrophic archaeon of interest to the biofuel community. However, current processes are not efficient enough to be industrially viable.^22,30^ To address this, Nichols and co-workers increased methane production by interfacing the metabolism of *M. barkeri* with a biocompatible water-splitting catalyst. The authors developed a two-compartment biocompatible electrochemical cell using a *n*-TiO_2_ photoanode and *p*-InP photocathode submerged in a culture of *M. barkeri* (Fig. 3B). Using solar energy, the microorganism converted CO_2_ and water to methane with high faradaic efficiency (74%) in minimal media, a 3-fold increase in methane production compared to unilluminated controls.^31^

More recently, Ye and co-workers increased methane production in *M. barkeri* using solar energy and a CdS nanoparticle catalyst (Fig. 3C).^32^ The authors hypothesised that photoexcitation of CdS would enable electron transfer to membrane-bound proteins cytochrome b and [NiFe] hydrogenase (Ech) in *M. barkeri*.^33^ After 3 days, a 13-fold increase in methane production was observed compared to controls cultured in the absence of CdS. Transcriptomic experiments showed a 1.5-fold increase in mRNA copies encoding *mcrA* within cells cultured in the electrochemical bioreactor. The *mcrA* gene encodes the α-subunit of methyl coenzyme M reductase, which catalyses the final step of methane biosynthesis. This demonstrates that a dynamic and cooperative interaction exists between the microbe and non-enzymatic catalyst during the reaction, and suggests that transcriptomic approaches may be used to guide the design of engineered hosts for new biocompatible reactions in the future.^32^ Further studies showed that the photocatalytic performance of the catalyst could be enhanced 3.5-fold by doping CdS with nickel. The authors hypothesised that nickel increases the stability of the nanoparticle under the reaction conditions, promoting the transfer of electrons from CdS* to *M. barkeri*.^33^

In addition to the generation of H_2_, Guo and co-workers recently used photogenerated electrons from InP nanoparticles to support cofactor regeneration in the yeast *Saccharomyces cerevisiae*.^34^ The knock-out strain *S. cerevisiae* Δ*zwf1* was used to impair NADP^+^ reduction in the cytosol, which could be rescued *via* non-enzymatic photoelectron transfer from InP located at the cell membrane. Illumination of cells resulted in a 35-fold increased NADPH-dependent reduction of dehydroshikimic acid (DHS) to shikimic acid (SA) and this continued for 72 h to exceed native SA yields from unmodified *S. cerevisiae* by 3-fold. Although the precise mechanism of electron transport from InP* to NADP^+^ is still under investigation, the increased redox activity of spent reaction cultures after *S. cerevisiae* Δ*zwf1* growth suggests the presence of additional redox-active species *in vivo*. Together this work highlights the use of biocompatible chemistry to complement and enhance native metabolic reactions in various microorganisms, and suggests a wider role of cellular metabolites in non-enzymatic catalysis in the cell interior.

## 3. Biocompatible chemistry for small molecule synthesis

### 3.1. Intracellular non-enzymatic catalysis

The examples discussed so far harness extracellular or cell surface mediated electron transfer processes to feed into microbial metabolism or abiotic polymer synthesis. A complementary form of biocompatible chemistry is the use of non-enzymatic catalysis to interface directly with native and engineered microbial metabolism to access reaction pathways that would not be accessible using synthetic chemistry or synthetic biology in isolation. If the catalyst can be generated by the microorganism or efficiently cross the cell membrane, this process may occur intracellularly. Whilst examples in this field are currently limited, there is considerable potential for this strategy to bridge native enzymatic pathways to enable new-to-nature metabolic transformations using both transition metal- and organocatalysis.^35^

**Fig. 4:**
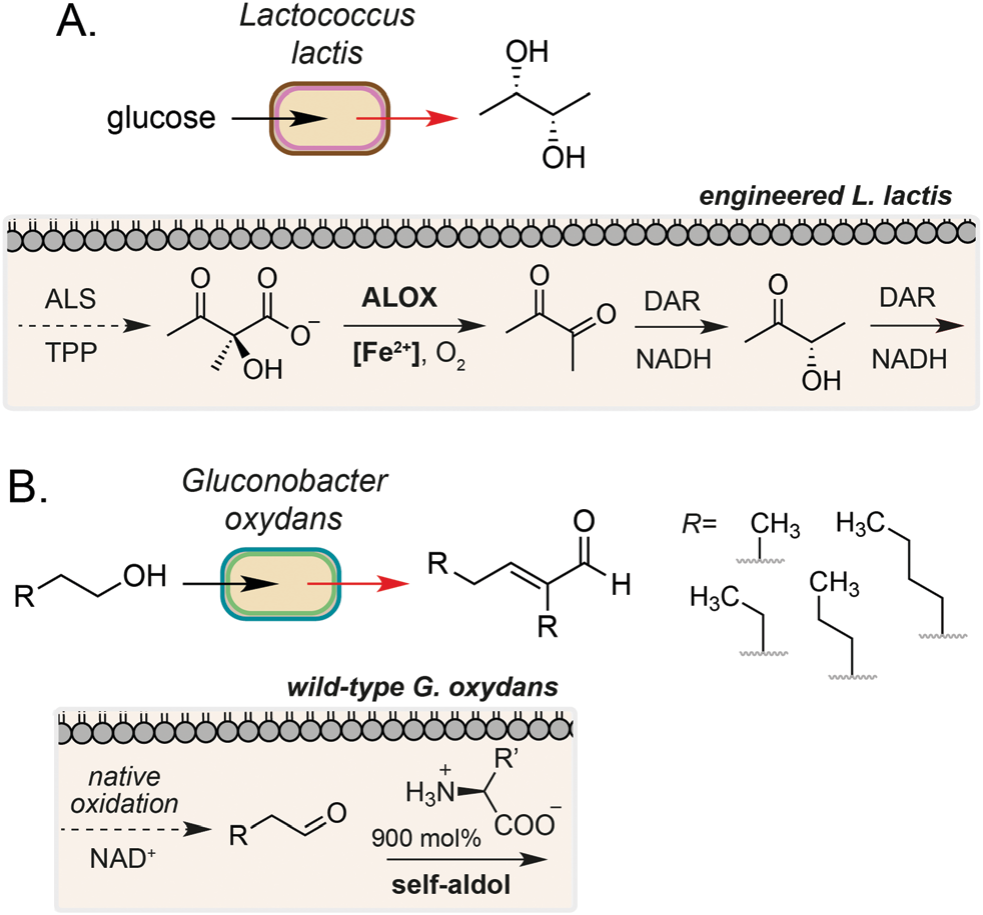
Intracellular non-enzymatic catalysis for production of high value small molecules. (A) Fe(ii)-catalysed non-enzymatic oxidative decarboxylation (ALOX) of acetolactate in *L. lactis* enables efficient access to diacetyl, acetoin and butanediol. DAR refers to diacetyl reductase from *Enterobacter cloacae*. (B) Native oxidation of short chain alcohols by *G. oxydans* coupled with an organocatalytic aldol condensation gives access to valuable α,β-unsaturated compounds.

A key example of intracellular biocompatible chemistry is the use of the Fe(iii) complex hemin to bridge two parts of an engineered biosynthetic pathway for high-yield production of the industrially valuable compounds diacetyl and (*S*,*S*)-2,3-butanediol by *Lactococcus lactis*.^36^ A knockout strain of *L. lactis* was generated to inactivate competing pathways and direct metabolic flux toward α-acetolactate formation. Hemin then catalysed the biocompatible non-enzymatic oxidative decarboxylation (ALOX) of α-acetolactate to form diacetyl, which could be reduced to (*S*,*S*)-2,3-butanediol by expression of a heterologous reductase. The bridging of these two pathways enabled redox-balanced and growth-coupled (*S*,*S*)-2,3-butanediol production under aerobic conditions (Fig. 4A). In a subsequent study, Liu and co-workers demonstrated extension of this system for production of (3*S*)-acetoin. In this work, a hemin-catalysed ALOX reaction was used to produce diacetyl which was coupled with a diacetyl reductase *in vivo* to achieve redox balancing and obtain high titres (5.8 g L^−1^) of the target compound.^37^

Beyond ALOX systems, many reported abiotic intracellular metal-catalysed reactions are examples of bioorthogonal, rather than biocompatible, chemistry. However, some of these systems do indirectly influence cellular metabolism, therefore serving as interesting examples of the interplay between biocompatible and bioorthogonal reactions *in vivo*. For example, bis[tri(2-furyl)phosphine]PdCl_2_ encapsulated in poly(lactic-*co*-glycolic acid)-*b*-polyethyleneglycol (“nano-Pd”) catalyses the uncaging of allyloxycarbonyl protected prodrugs and can trigger tumour cell death by release of the active agent, which interferes with cell replication processes.^38^ In a second example, an intracellular bis-allyl Ru(iv)-catalysed redox isomerisation of allylic alcohols led to the accumulation of α,β-unsaturated ketones, which deplete intracellular glutathione levels *via* trapping in a Michael addition reaction.^39^ In both examples, however, the key difference from biocompatible systems is that the substrate for non-enzymatic catalysis is exogenous and the product does not feed into cellular metabolism.

A complementary approach to metal-catalysed biocompatible chemistry is interfacing microbial metabolism with organocatalysis. In 2016, Domaille and co-workers demonstrated an amino acid-catalysed aldol condensation reaction that was compatible with *E. coli* growth conditions.^40^ A range of short-chain aldehydes could be converted into the corresponding self-aldol condensation product at room temperature in bacterial growth media using β-alanine and other biogenic amines as an organocatalyst. Addition of glyceryl tributyrate enabled yields of up to 70% and improved cell viability by alleviating toxicity of the α,β-unsaturated products by *in situ* product removal (ISPR). The reaction products are also industrially valuable; the self-aldol condensation product of butanal dimerization is a key intermediate in the synthesis of 2-ethyl-2-hexanol, which is currently produced on multimillion-tonne scale annually using Rh catalysis from fossil fuel derived feedstocks. In a subsequent study, Stewart and co-workers showed that the aldehyde substrate could be produced *via* the oxidation of primary alcohols by wild-type *Gluconobacter oxydans*.^41^ Coupling this with a lysine organocatalyst at 900 mol% provided the target α,β-unsaturated products in up to 88% yield over 24–48 h, redirecting metabolism away from further oxidation of the aldehyde to the carboxylic acid by native dehydrogenases. A range of cross-aldol products could also be obtained through addition of other C_2_–C_6_ alcohol substrates (Fig. 4B). Although the precise location of the biocompatible reaction in *E. coli* and *G. oxydans* was not confirmed in either study, primary alcohol oxidation in *G. oxydans* is known to occur at the cytosolic membrane^42,43^ and amino acid importers are known in both microorganisms, suggesting that organocatalysis could occur in the periplasmic space. However, the absence of any enhanced rate in the presence of cells due to substrate/catalyst co-localisation and the high catalyst loading required for product formation *in vivo* suggests that organocatalysis, in this case, likely occurs in the extracellular media after export of the aldehyde.

This work is an important example of using organocatalysis for biocompatible chemistry and lays the foundation for future development of more efficient systems and novel synthetic pathways. For example, Brewster and co-workers have recently demonstrated that α,β-unsaturated compounds such as 2-ethylhexenal can undergo enantioselective C=C bond reduction in the presence of unmodified *E. coli*.^44^ Since bio-based routes to *n*-butanol are known,^45–48^ it is therefore possible to envisage a system for the bio-based synthesis of 2-ethyl-2-hexanol using microbial cell factories.^44^ Further to enabling production of high value products under very mild processing conditions, these studies also illustrate the potential of unmodified microorganisms to generate substrates for biocompatible reactions. Whilst the study of biocompatible chemistry inside living cells is still in its infancy, these examples set a precedent for the development of powerful and elegant bio-based routes to high value small molecules using low cost small molecule catalysts.

### 3.2. Extracellular non-enzymatic catalysis for small molecule synthesis

Microorganisms have evolved sophisticated mechanisms to control the entry of abiotic molecules into the cell interior.^49,50^ Whilst essential for cell survival in native environments, this can present as a major challenge when engineering novel pathways using biocompatible chemistry. Conversely, using extracellular non-enzymatic catalysis to modify metabolites obviates the requirement for small molecule catalysts to accumulate within the cell. Many metabolites are also excreted from the cell, such that extracellular modification is preferable for maximum efficiency. Additionally, modification of metabolites can be an effective method of driving flux through a metabolic pathway towards the product of interest.^51^ Furthermore, microbes are able to alter the chemistry of their external environment (*e.g.* pH and/or O_2_ levels^52^), potentially facilitating non-enzymatic reactions. These factors make extracellular non-enzymatic catalysis an attractive strategy in many biotechnological applications.

**Fig. 5:**
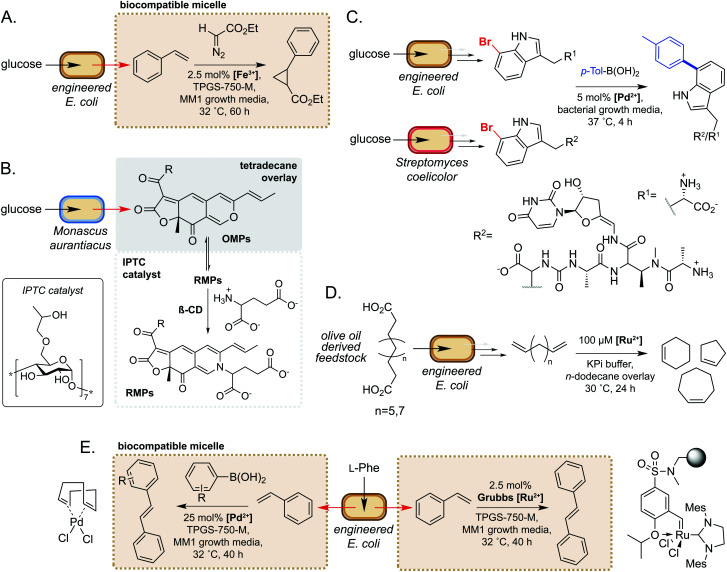
Extracellular non-enzymatic biocompatible catalysis. (A) Designer micelles increase flux through an engineered styrene pathway from d-glucose and can be interfaced with iron-catalysed cyclopropanation. (B) Inverse phase transfer catalysis accelerates the reaction between lipophilic OMPs and hydrophilic glutamate to produce high value OMPs. (C) The ‘GenoChemetics’ platform couples microbial production of non-natural brominated natural products with extracellular Pd-catalysed cross-coupling to generate abiotic molecules. (D) Generation of high value cycloalkenes *via* interfacing biocatalytic decarboxylation of olive oil derived feedstocks with Ru-catalysed ring-closing metathesis. (E) Use of biocompatible micelles to interface a microbial styrene production pathway from l-Phe with Pd- and Ru-catalysis to generate stilbene and stilbene derivatives.

A seminal example from Wallace and Balskus in 2016 is the use of biocompatible vitamin E-derived dl-α-tocopherol polyethylene glycol succinate (TPGS) micelles to interface styrene production in *E. coli* with Fe(iii)-catalysed cyclopropanation (Fig. 5A).^51^ The micelles were shown to associate non-covalently with the bacterial outer membrane, which was hypothesised to increase membrane permeability and increase the rate of styrene removal from the cell cytosol. This ISPR strategy increased flux through the pathway, leading to a 2-fold increase in cyclopropane product titres whilst also mitigating the toxicity of styrene to *E. coli*. This study was the first example of micellar catalysis in the presence of a living cell and has prompted considerable interest in exploring further applications of biocompatible micelles and ISPR.^53–56^

In a second example, Gu and co-workers used inverse phase transfer catalysis to facilitate production of red *Monascus* pigments (RMPs) from fermentation derived orange *Monascus* pigments (OMPs) (Fig. 5B). In their model system, a biphasic aqueous-tetradecane system was established using a β-cyclodextrin derivative as an inverse phase transfer catalyst (IPTC) to accelerate the reaction between the lipophilic OMPs and hydrophilic glutamate.^57^ Directly interfacing a fermentation of the mould *Monascus aurantiacus* with this system enabled product titres of almost 2 g L^−1^, a 6- and 1.5-fold increase on controls lacking the IPTC catalyst and tetradecane, respectively. Furthermore, this approach gave high reaction selectivity for RMPs and very low levels of OMPs and side-products from competing pathways. These examples demonstrate the potential of compartmentalising microbial metabolites to enable biocompatible chemistry to take place.

There have also been notable examples of interfacing microbial metabolism with aqueous phase, extracellular transition metal catalysis. In 2017, Sharma and co-workers reported a ‘GenoChemetics’ platform in which halide tagged metabolites could undergo Pd-catalysed Suzuki–Miyaura cross-coupling *in situ* to generate non-natural analogues.^58^ In the first model system, 7-bromotryptophan produced by an engineered strain of *E. coli* was cross-coupled in a one-pot process with *p*-tolylboronic acid, whilst a second example demonstrated production and subsequent cross-coupling of a brominated derivative of the antibiotic pacidamycin from *Streptomyces coelicolor* (Fig. 5C). Further to applications in chemical synthesis, this platform may have uses in directed evolution assays and studying the fate of tagged natural products in biological systems.^58^

More recently, Wu and co-workers developed a one-pot process for the conversion of oleic acid to cycloalkenes, bulk petrochemicals which are widely used in industrial processes.^59^ The membrane bound desaturase-like enzyme UndB from *Pseudomonas sp.* was found to efficiently catalyse the bis-decarboxylation of azelaic, sebacic and undecanedioic acids when heterologously expressed in *E. coli*. The resultant bis-alkene products could then undergo biocompatible Ru-catalysed cyclization *via* ring-closing metathesis in an aqueous-*n*-dodecane two-phase system. This enabled access to cyclopentene, cyclohexene and cycloheptene from olive oil derived feedstocks, unlocking a new area of chemical space in the field of synthetic biology (Fig. 5D). In a related study, Maaskant and co-workers reported use of the biocompatible micelle strategy to merge microbial styrene production with (i) Ru-catalysed metathesis and (ii) Pd-catalysed cross-coupling with a boronic acid.^56^ For the metathesis reaction, use of an immobilised Zhan-II Ru catalyst enabled ∼30% overall conversion from phenylalanine to stilbene *via* styrene produced by engineered *E. coli.* The addition of 0.25% w/v or 2.5% w/v biocompatible micelles led to 2- or 3-fold increase in styrene titres, respectively. Interestingly, however, the addition of micelles did not lead to an increase in stilbene yields, which was hypothesised to be due to sequestration of styrene by the hydrophobic immobilisation resin. For the Pd-catalysed cross-coupling methodology, yields of ∼30% were obtained *via* a two-step process comprising initial production of styrene by engineered *E. coli*, followed by addition of the Pd(COD)Cl_2_ catalyst, micelles and boronic acid. This methodology was further extended to the cross-coupling of styrene with a panel of substituted arylboronic acids (Fig. 5E). These studies are excellent examples of the power of biocompatible chemistry in enabling overall chemical transformations that would not be accessible using synthetic biology or synthetic chemistry in isolation and unlocks exciting possibilities to access new areas of chemical space from renewable feedstocks.

Extracellular non-enzymatic chemistry has also been demonstrated with other small molecule catalysts. In 2020, Liu and co-workers reported a phosphate-catalysed biocompatible reaction to produce azaphilone alkaloids, a group of fungal polyketides with applications in drug discovery and as food colorants.^60^ Phosphate (added as KH_2_PO_4_) was employed as a Brønsted acid catalyst in the presence of *Penicillium sclerotiorum* to enable the one-pot conversion of fermentation-derived azaphilone into the corresponding alkaloid *via* reaction with a primary amine. This method enabled rapid access to a library of non-natural azaphilone alkaloids under very mild conditions and was also hypothesised to alleviate product inhibition of the polyketide pathway.^60^

Finally, it is also noteworthy that a 96-well plate assay has been developed to rapidly identify biocompatible transition metal catalysts.^61^ This system couples biocompatible catalyst activity with a fluorescence readout *via* incorporation of an *in situ* synthesized non-canonical amino acid into green fluorescent protein through Amber stop-codon suppression, a strategy that has also been used to ‘addict’ *E. coli* to abiotic reactions.^62^ This methodology was hypothesised to be widely applicable to any chemical transformation that yields a non-canonical amino acid and therefore presents a general platform for the identification of new biocompatible reactions in the future.

## 4. Conclusions and outlook

This review has highlighted recent examples (2014-present) of non-enzymatic catalysis interfaced with cellular metabolism to produce valuable non-natural chemicals and materials. In this short time, there has been remarkable progress in this young field, uniting the two traditionally discrete fields of synthetic chemistry and synthetic biology. Unmodified and engineered microbes can mediate redox processes for polymer synthesis and show biocompatibility with solar-light driven photochemical reactions, whilst non-enzymatic catalysts can be employed both intra- and extracellularly to access new-to-nature areas of chemical space. In an ongoing drive towards a more sustainable future, synthetic chemistry reactions are increasingly being demonstrated in aqueous conditions at ambient temperatures. In tandem, synthetic biologists continue to engineer microbes to valorise renewable and waste feedstocks and show resilience towards harsher processing conditions. Together with a better understanding of how to interface chemical and biological systems such as the use of biocompatible micelles and biphasic systems, we believe that this field holds exciting potential to deliver sustainable processes for the chemical industry.

## Conflicts of interest

There are no conflicts to declare.

## Supplementary Material
